# Construction and Verification of a Glycolysis-Associated Gene Signature for the Prediction of Overall Survival in Low Grade Glioma

**DOI:** 10.3389/fgene.2022.843711

**Published:** 2022-03-23

**Authors:** Wei Liu, Chunshan Liu, Chengcong Chen, Xiaoting Huang, Qi Yi, Yunhong Tian, Biao Peng, Yawei Yuan

**Affiliations:** ^1^ Department of Radiation Oncology, Affiliated Cancer Hospital and Institute of Guangzhou Medical University, Guangzhou, China; ^2^ Department of Neurosurgery, Affiliated Cancer Hospital and Institute of Guangzhou Medical University, Guangzhou, China

**Keywords:** low-grade glioma, glycolysis, signature, prognosis, risk model

## Abstract

The overall survival of patients with lower grade glioma (LGG) that might develop into high-grade malignant glioma shows marked heterogeneity. The currently used clinical evaluation index is not sufficient to predict precise prognostic outcomes accurately. To optimize survival risk stratification and the personalized management of patients with LGG, there is an urgent need to develop an accurate risk prediction model. The TCGA-LGG dataset, downloaded from The Cancer Genome Atlas (TCGA) portal, was used as a training cohort, and the Chinese Glioma Genome Atlas (CGGA) dataset and Rembrandt dataset were used as validation cohorts. The levels of various cancer hallmarks were quantified, which identified glycolysis as the primary overall survival-related risk factor in LGGs. Furthermore, using various bioinformatic and statistical methods, we developed a strong glycolysis-related gene signature to predict prognosis. Gene set enrichment analysis showed that in our model, high-risk glioma correlated with the chemoradiotherapy resistance and poor survival. Moreover, based on established risk model and other clinical features, a decision tree and a nomogram were built, which could serve as useful tools in the diagnosis and treatment of LGGs. This study indicates that the glycolysis-related gene signature could distinguish high-risk and low‐risk patients precisely, and thus can be used as an independent clinical feature.

## Introduction

Glioma is the most common intracranial malignant tumor, which accounts for 80% of all intracranial malignancies, with 15,000–17,000 new cases every year in America (Ostrom et al., 2018). The World Health Organization defined grade II and III gliomas as diffuse lower grade gliomas (LGGs), occupying approximately 30% of intracranial tumors (Brat et al., 2015). LGGs predict a better prognosis than glioblastoma; however, despite their highly heterogeneous natural processes, most LGGs progress to aggressive high-grade secondary gliomas that ultimately cause death (Zhang et al., 2020). Despite great advances in treatment for LGGs being achieved, including neurosurgery, radiotherapy, and chemotherapy, the treatment of LGGs remains a challenge (Youland et al., 2017). Recently, our ability to diagnose and prognose LGGs has been enhanced by the discovery of various biomarkers. The classification of CNS tumors was revised by the World Health Organization in 2016, based on morphological and molecular features, which highlighted the importance of molecular testing and signature construction for the diagnosis and prognosis of LGGs (Louis et al., 2016).

Compared with healthy cells, cancer cells have very different metabolic patterns, adjusting metabolism to sustain the biosynthetic demands of tumor proliferation and have a higher resistance to cell death pathways (Abbaszadeh et al., 2020). Changes in the tumor glucose metabolic environment are often associated with oxygen supply and demand, dysfunction of key enzymes, or mitochondrial dysfunction, frequently resulting in advanced tumor aggressiveness, poor prognosis, and limited efficacy of available therapeutic methods (Ancey et al., 2018). Although some studies have shown that tumor glucose metabolism disorders are closely associated with cancer progression and low survival in gliomas, there is no glycolysis-based approach to distinguish high-risk patients (Gabriely et al., 2017; Strickland and Stoll, 2017).

There has been significant progress in molecular markers; however, they are still not satisfactory and there is still much room for improvement (Zhang et al., 2017). The present study aimed to determine whether glycolysis is a primary survival-related risk factor for LGGs, to identify powerful biomarkers, and construct a glycolysis-related gene signature for LGG prognosis. Furthermore, the prognostic efficiency of the model was tested in two additional validation sets. Finally, we established a comprehensive model based on clinicopathological and genetic characteristics to enhance the signature’s accuracy and predictive power, which might be applied to guide the clinical care and patient consultation.

## Materials and Methods

### Preparation and Processing of Data

We downloaded the transcriptome profiles (HTSeq Fragments Per kilobase of transcript per Million mapped reads [FPKM]) and their associated clinical profiles from TCGAbiolinks (Colaprico et al., 2016), which was used as The Cancer Genome Atlas (TCGA) training cohort (*n* = 501) to establish the risk score signature. The *IDH1* (encoding isocitrate dehydrogenase 1) statuses were calculated using maftools (Mayakonda et al., 2018). The first validation cohort comprised RNA sequencing (RNA-seq) data from the Chinese Glioma Genome Atlas (CGGA) database (*n* = 552). Gene expression was also normalized and calculated using the FPKM method. Rembrandt microarray dataset was downloaded from CGGA database and used as the second validation cohort (*n* = 123) (Gusev et al., 2018). The 13,609 common genes in 3 independent cohorts are used for the following analysis.

The batch effect of RNA-seq data in the CGGA database, which comprised two independent cohorts, was removed using SVA packages (Brägelmann and Lorenzo Bermejo, 2019). Patients with grade II and III glioma with complete sex, age, *IDH1* status, and survival information were used in the following study. The patients with grade II and III glioma in the Rembrandt dataset with complete survival information were used for the follow-up study. For all the included RNA-seq and microarray data, normalization and log2 transformation were performed. The RNA-seq data were adopted in FPKM methods, the microarray data were normalization using RMA methods by oligo package (Carvalho and Irizarry, 2010).

### Screening Candidate Genes and Building the Signature

The RNA-seq training data and hallmark gene sets downloaded from the Molecular Signatures database (MSigDB) (Liberzon et al., 2015) were used by a single-sample gene set enrichment analysis (ssGSEA) algorithm (Hänzelmann et al., 2013) as the basis to calculate the quantified score of each cancer hallmark. Univariate Cox analysis was used to calculate the significance of different cancer hallmarks in patients with LGG. With a network type of unsigned and a soft threshold of *β* = 5 (scale free *R* ^2^ = 0.8997766), the expression values of protein coding genes in the LGG samples were subjected to weighted gene co-expression network analysis (WGCNA) to construct a scale-free co-expression network, which was used to screen those genes that were most correlated with glycolysis, based on their ssGSEA scores (Langfelder and Horvath, 2008). Subsequently, 19 modules were identified by setting the merged threshold function at 0.25, the green module was identified the genes of significantly related module. The correlation between the gene expression profiles and module eigengenes was measured using module membership (MM), and the correlation between the glycolysis ssGSEA score and individual genes represented the gene significance (GS). We screened 407 extracted candidates from the “glycolysis module” using a cutoff *p* value of GS of <0.0001 and the univariate Cox regression calculated *p* value of <0.01. Then, a least absolute shrinkage and selection operator (Lasso) Cox regression algorithm were employed to identify the most significant prognostic markers (Friedman et al., 2010). We then constructed a glycolysis-related risk score (GRS) that included normalized gene expression values weighted by their LASSO Cox coefficients as follow:

$$GRS=\overset{n}{\underset{i=1}{\sum }}coefficient\left(mRNA\text{i}\right)*expr\left(mRNAi\right)$$

### Gene Ontology (GO) and Kyoto Encyclopedia of Genes and Genomes (KEGG) Enrichment Analysis Based on DEGs Between High GRS Score and Low GRS Score

Based on median value of GRS score, we divided the patients in training cohort into high-risk group and low-risk group, we obtain 1827 differentially expressed genes (*p* < 0.05, |log_2_FC|>1) using the edgeR (Robinson et al., 2010) package of R between two group. Based on the differential genes that have been obtained, Gene ontology and Kyoto encyclopedia of genes and genomes enrichment analyses were launched to probe the potential biological functions and signaling pathways by clusterProfiler (Yu et al., 2012), enrichplot packages (Yu, 2021). Standards of significantly enriched terms were set as follow: q-value < 0.05.

### Gene Set Enrichment Analysis

We utilized the msigdb. v7.0. entrez.gmt gene sets from the MsigDB database and clusterProfiler packages to explore the therapeutic resistance and possible cellular pathways. We set following standards for significantly enriched terms: I. NOM *p*-value < 0.05; II. FDR q-value < 0.05.

### Drug Sensitivity of the Members in GRS Score

The drug sensitivity of the members in GRS score were calculated by online webtool GSCALite (http://bioinfo.life.hust.edu.cn/web/GSCALite/). The results from CTRP database were adopt and displayed in our study.

### Development and Evaluation of Clinical Predictive Models

A decision tree was constructed using recursive partitioning analysis (RPA) in the “rpart” package for risk stratification (Terry and Beth, 2019). For the quantitative prediction of the prognosis of patients with LGG, we established a prognostic nomogram, comprising the glycolysis-based risk model and other clinical parameters, which was used to predict the probability of overall survival (OS) for 1, 3, and 5 years. The predictive ability of the model was demonstrated by plotting a calibration curve, in which a curve close to 45° indicates a good predictive ability.

### Bioinformatic and Statistical Analyses

Data processing and graph construction were carried out using R software (version 4.0.3, http://www.r-project.org). Adobe Illustrator 2020 was used to fine tune the graphics. Survival was evaluated using the Kaplan-Meier method. The differences between the high-risk group and low-risk group in the targeted cohorts were compared using a Log-rank test. To evaluate the risk signature’s predictive performance, receiver operating characteristic curve (ROC) and area under the curve (AUC) at 1, 3, and 5 years were calculated in all cohorts using the “timeROC” packages (Blanche et al., 2013). In the absence of a full-scale gene signature expression profile, each cohort was divided into different clusters according to the optimum k value using K-means consensus clustering in the “ConsensusClusterPlus” packages, The optimal cluster numbers were determined by constructing CDF curves respectively (Wilkerson and Hayes, 2010). In the pooled cohort, to assess the prognostic value, a meta-analysis (I^2^ = 97%, random-effect model) was used.

## Results

### Schematic Diagram of Research Design

**FIGURE 1:**
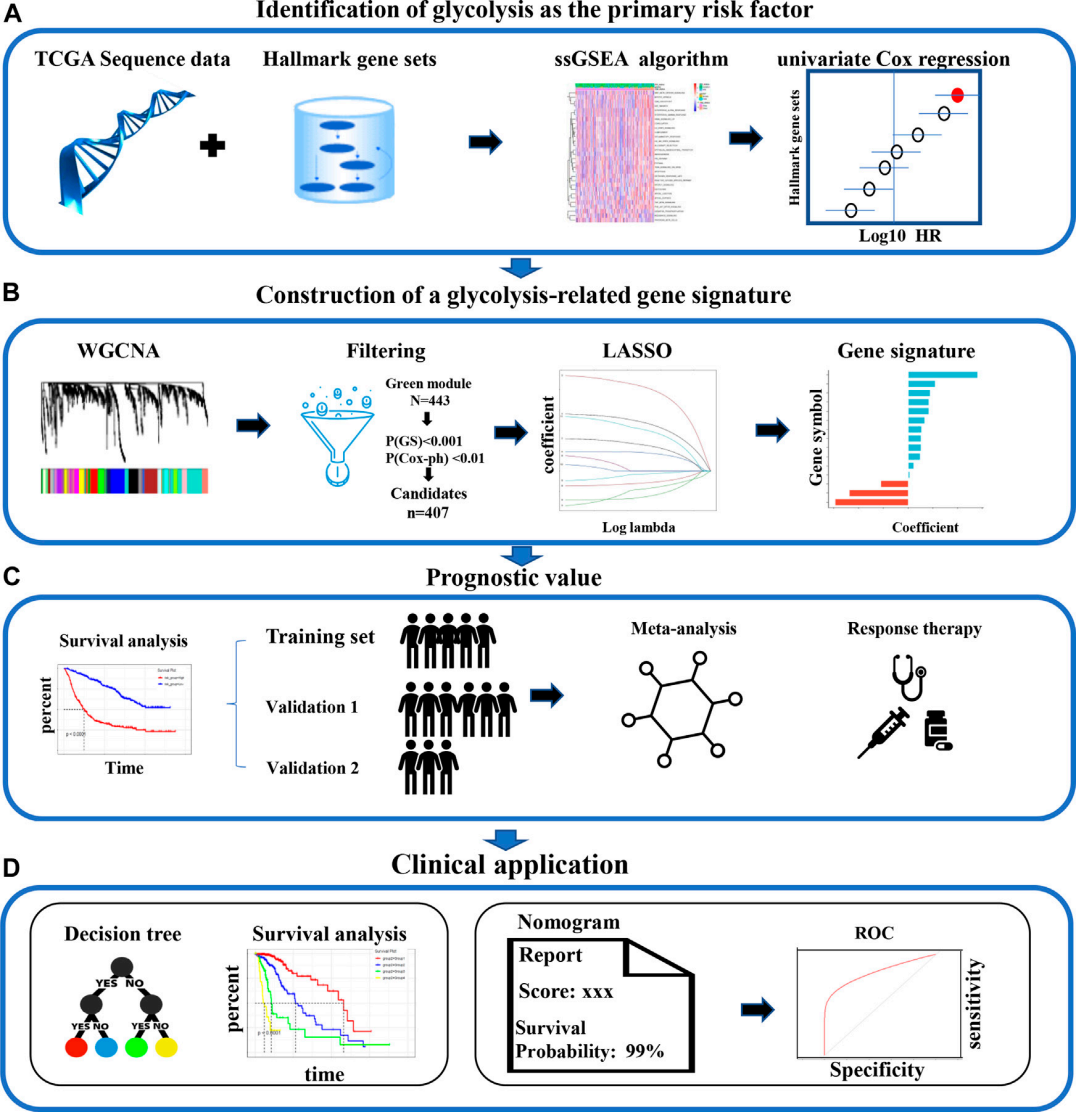
The analysis the study workflow. **(A)** Among various hallmarks of cancer, we identified glycolysis as the primary risk factor for OS in patients with LGG **(B)** A robust glycolysis-related gene signature for patients with LGG was constructed using various bioinformatic methods. **(C)** The validation of the gene signature’s prognostic value in the CGGA and Rembrandt cohorts **(D)** Clinical significance. LGG: low-grade glioma; WGCNA, weighted gene co-expression network analysis; LASSO, least absolute shrinkage and selection operator; ROC, receiver operating characteristic; OS: overall survival.

The information for the patients in each cohort who met the inclusion criteria is listed in Supplementary Table S1. First, among the various hallmarks of cancer, we identified glycolysis as the primary OS-related risk factor in patients with LGG (Figure 1A). Then, WGCNA, univariate Cox regression analysis, and the LASSO algorithm were used to screen candidate genes and construct a robust signature, which could be used to predict patient survival reliably (Figure 1B). Next, the glycolysis signature’s prognostic efficiency was assessed in the training set and two independent validation datasets. In addition, to confirm the signature’s prognostic accuracy, a meta-analysis was preformed, and the usability of the signature in clinical practice was assessed by choosing the response to therapy as an important evaluation indicator (Figure 1C). Finally, we built a decision tree for prognostic precision improvement, and constructed a nomogram based on GRS and other clinicopathological indicators to evaluate the level of risk and the individual patients’ survival probability (Figure 1D).

### Glycolysis Is the Primary OS-Related Risk Factor in LGGs

**FIGURE 2:**
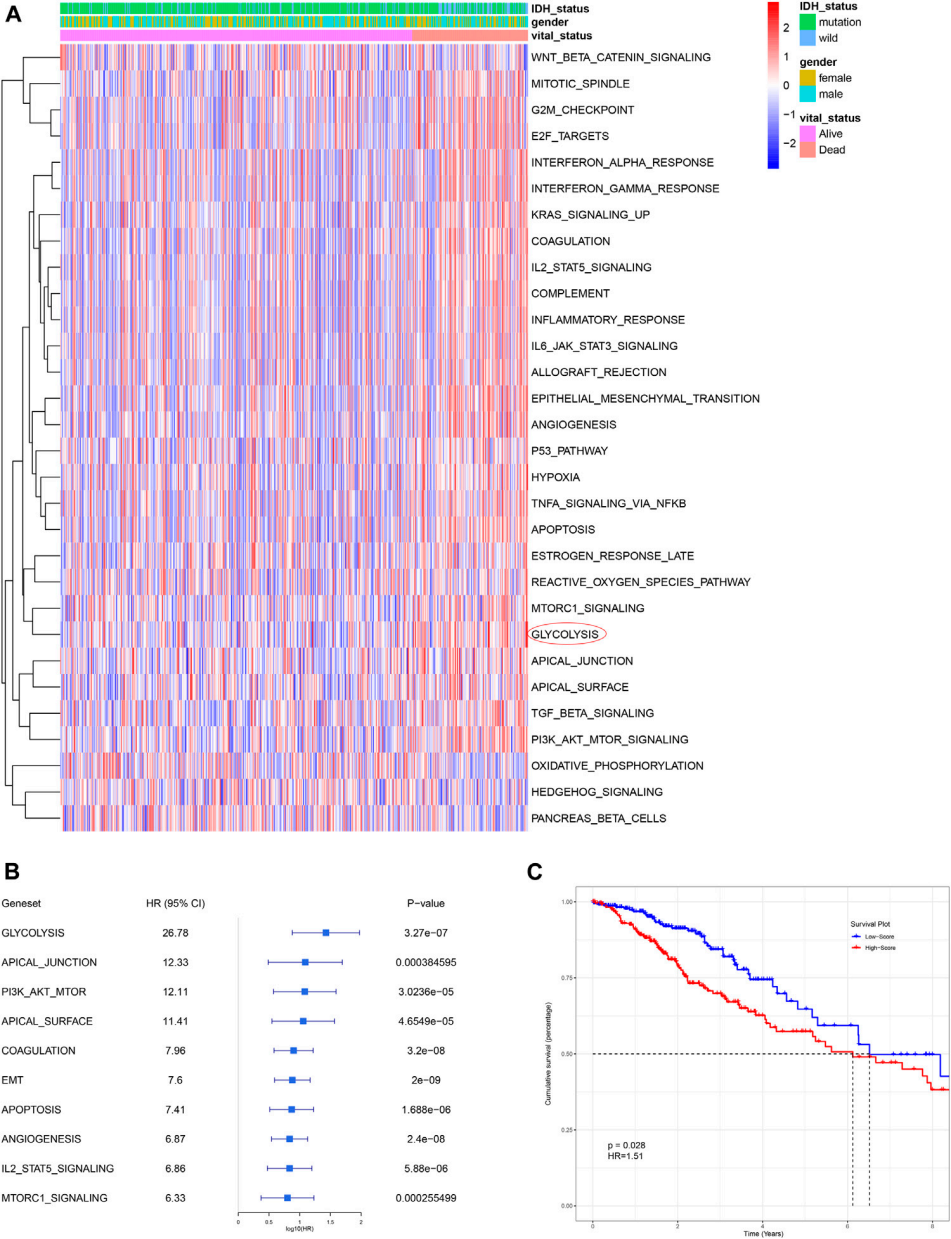
Glycolysis was identified as the primary survival-related risk factor. **(A)** Heatmap for different hallmark pathway enrichment scores generated by the ssGSEA algorithm. **(B)** Univariate Cox analysis of top 10 hallmark pathways. **(C)** Patients with a higher glycolysis-associated ssGSEA score had a worse OS, as shown by Kaplan-Meier analysis. OS: overall survival.

ssGSEA is an analytical algorithm that uses RNA expression data to score different cancer hallmarks in each sample. The score of 50 hallmark gene sets were calculated and 30 gene sets with a *p*-value < 0.05 were considered to represent significantly enriched pathways (Figure 2A). Compared with other cancer gene sets, such as those representing apical junction, apoptosis, and angiogenesis, the glycolysis-related gene set had the most powerful influence (highest HR) on survival (Figure 2B). Based on their median ssGSEA score, patients (*n* = 501) could divide into a low score group and a high score group, which exhibited poorer OS compared with those in the low score group (*p* < 0.05; Figure 2C).

### Construction of a Prognostic Glycolysis-Related Gene Signature

**FIGURE 3:**
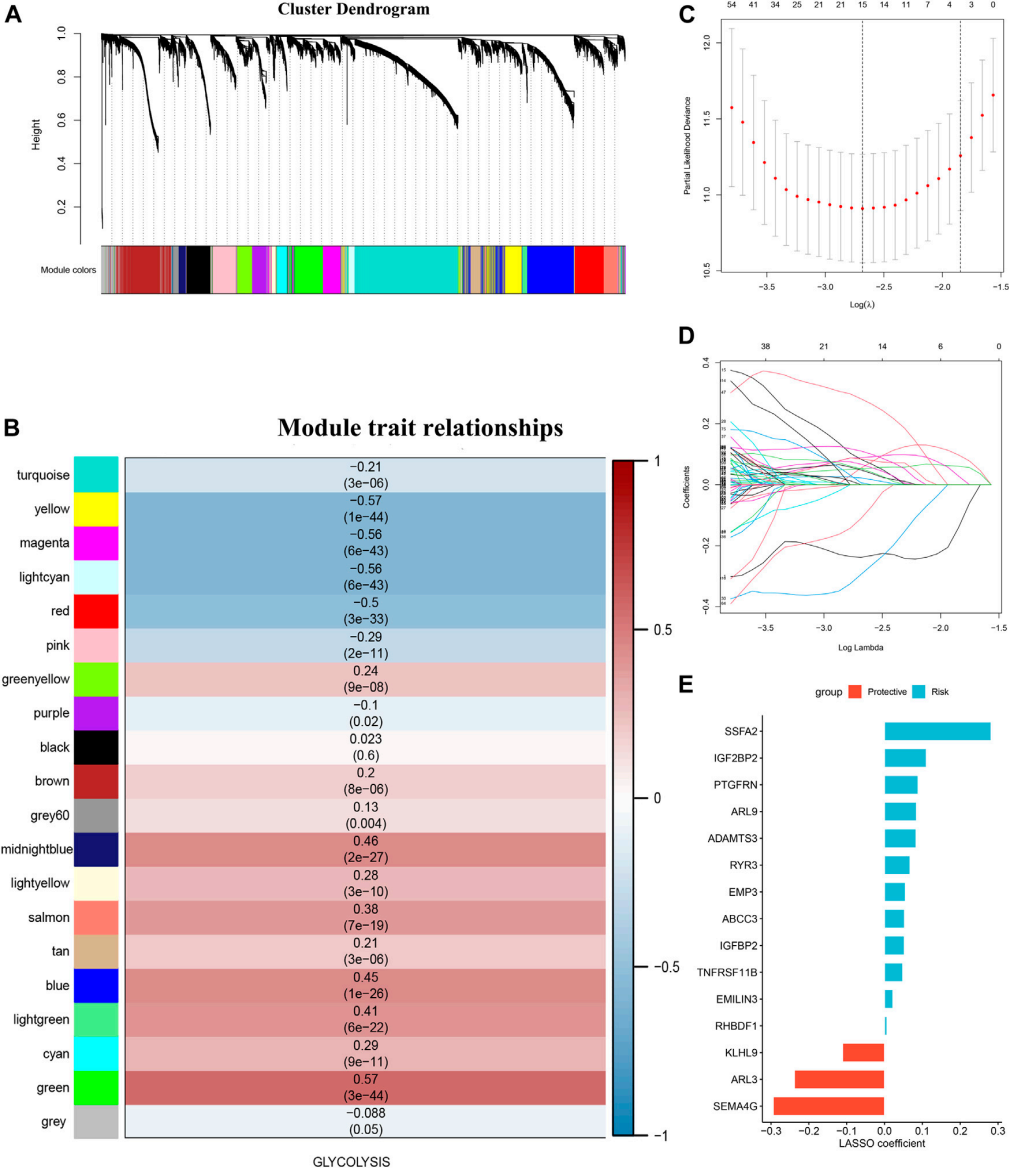
Glycolysis-associated gene signature construction. **(A)** was perform with the top 50 percent of variant genes commonly expressed in the training and testing cohorts were subjected to WGCNA **(B)** Gene modules associated with the glycolysis enrichment score obtained using WGCNA. **(C)** Cross-validation plot for the penalty term. **(D)** Plot of the LASSO expression coefficients of 15 glycolysis-related genes. **(E)** LASSO coefficients of the glycolysis-related gene signature.

The top 50 percent of differentially expressed genes (6,805 genes), selected on the basis of an analysis of variance and their glycolysis ssGSEA score in the training set, were chosen for WGCNA. The optimal soft-thresholding power was 5, which ensured that the co-expression network was scale free (Supplementary Figure S1A). Specifically, 20 co-expression modules were identified after merging modules with similarities above 0.75 (Figure 3A). To assess the stability of each module identified in the training cohort, we divided the TCGA data into training and testing cohort to conduct module preservation analysis using the module preservation test (nPermutations = 200) in the WGCNA package; modules with Z-score >10 were considered to be strongly preserved (Supplementary Figure S1B). Among the modules with Z-score >10, the green module showed the highest correlation (r = 0.57, p = 3e-44) and was thus considered to correlate the most with glycolysis (Figure 3B). Using a *p* value <0.0001 as the filtering criterion for GS, univariate Cox regression analysis was performed on hub genes extracted from the green module. Based on a threshold *p* value < 0.01 for the univariate Cox regression analysis, 407 candidate genes were submitted to the LASSO Cox regression algorithm, which can identify the strongest prognostic markers for prognosis. Ten-fold cross-validation was used to abrogate over-filtering, by selecting an optimal λ value of 0.0684 (Figure 3C). Fifteen genes (*ARL3*, *EMP3*, *IGFBP2*, *PTGFRN*, *ADAMTS3*, *ARL9*, *SEMA4G*, *RYR3*, *TNFRSF11B*, *SSFA2*, *ABCC3*, *EMILIN3*, IGF2BP2, *KLHL9*, and *RHBDF1*) retained their individual non-zero LASSO coefficients (Figure 3D). The LASSO coefficients of each gene in the GRS. Thus, we established the GRS formula, among the members of the signature, *KLHL9*, *ARL9* and *SEMA4G* are protective factors, the others are risk factors (Figure 3E and Supplementary Table S2).

### GRS Is a Risk Factor for OS in Each Cohort

**FIGURE 4:**
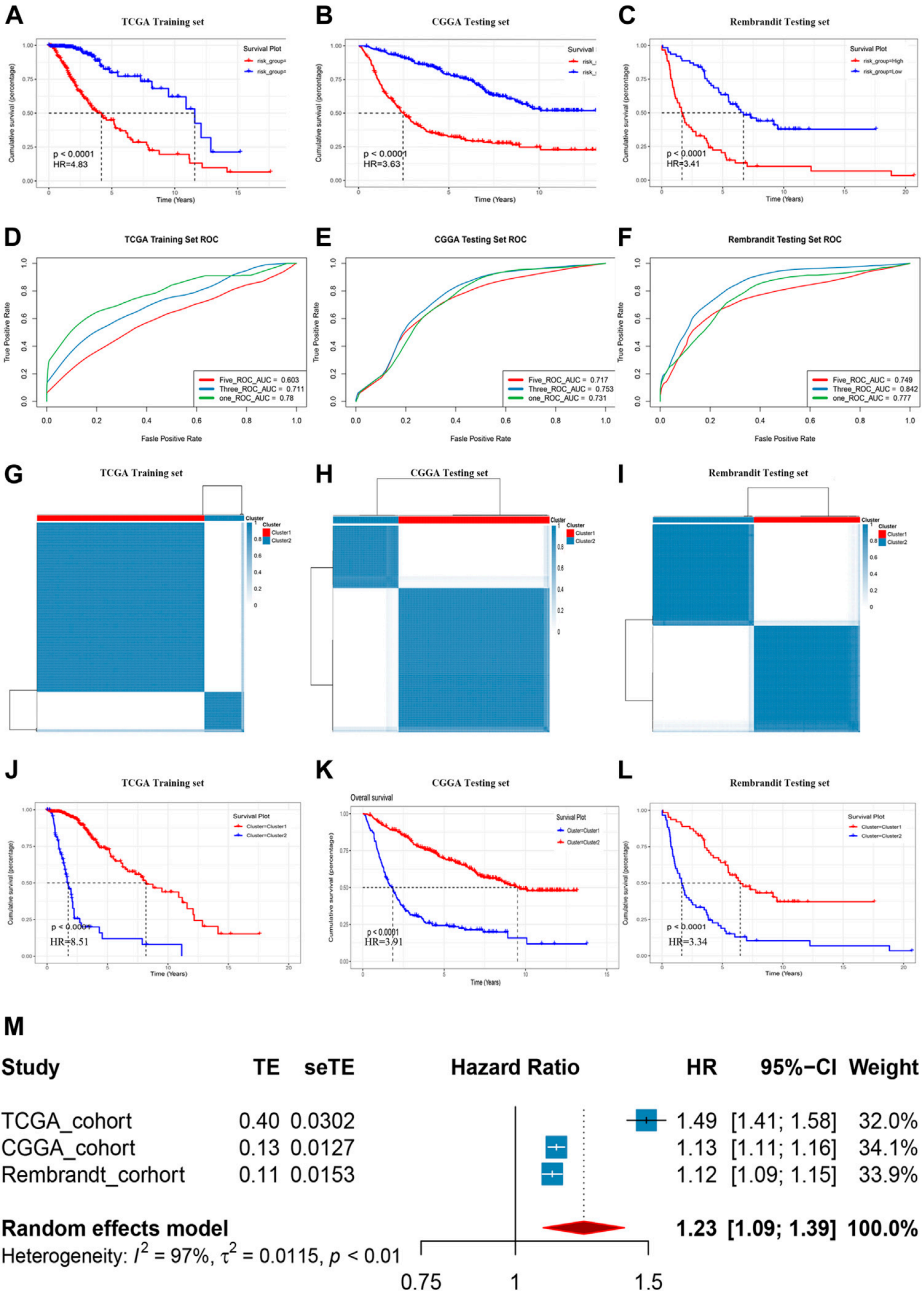
Prognostic risk score features of glycolysis-related genes in the training and testing cohorts. **(A-C)** Kaplan–Meier survival curve in the Rembrandt cohort, CGGA cohort, and TCGA cohort. **(D-F)** ROC curves of the GRS on OS in the three cohorts. **(G-I)** Sample clustering heat maps of the three cohorts generated using the K-mean cluster algorithm. **(J-L)** Kaplan–Meier survival curve in respectively clusters generated by K-mean cluster algorithm in three cohorts. **(M)** Meta-analysis of training cohort and testing cohorts.

The median GRS risk score in the training set and two validation sets were used to categorize patients with LGG into low- and high-risk groups. In each cohort, patients in the high-GRS score group had worse OS than those with a low GRS score (Figures 4A–C, *p* < 0.0001). Time-dependent ROC curves were used to evaluate the reliability of the GRS (Figures 4D–F). The AUC values were 0.78, 0.711, and 0.603 for 1-year, 3-years, and 5-years survival, respectively, in the TCGA training set, implying good reliability of the GRS to monitor survival. In the CGGA validation cohort, the AUC values were 0.731, 0.753, and 0.717; and in the Rembrandt cohort, the AUC values were 0.777, 0.842, and 0.749 for 1-year, 3-years and 5-years survival, respectively. Clustering was performed using a k-means algorithm which divided each cohort into different groups based on the best k value of genes in the GRS signature (Figures 4G–I), basing on the cumulative distribution function (CDF) curves (Supplementary Figure S2A: TCGA, Supplementary Figure S2B: CGGA, Supplementary Figure S2C: Rembrandt). The results showed that the OS differed significantly among the k-means derived groups, which suggested that the classification means based on genes in the signature could be used directly for tumor subtyping (Figure 4J-L). Moreover, a meta-analysis of the prognostic value of the GRS in the pooled cohort, which integrated the TCGA training cohort and the two validation cohorts, showed that among all the 1,176 patients in three cohorts, those with a lower GRS had a better prognosis than those with a high GRS (pooled HR = 1.23, 95% confidence interval (CI): 1.09–1.39; Figure 4M).

### GRS Score Was Associated With the Clinicopathological Characteristics

**FIGURE 5:**
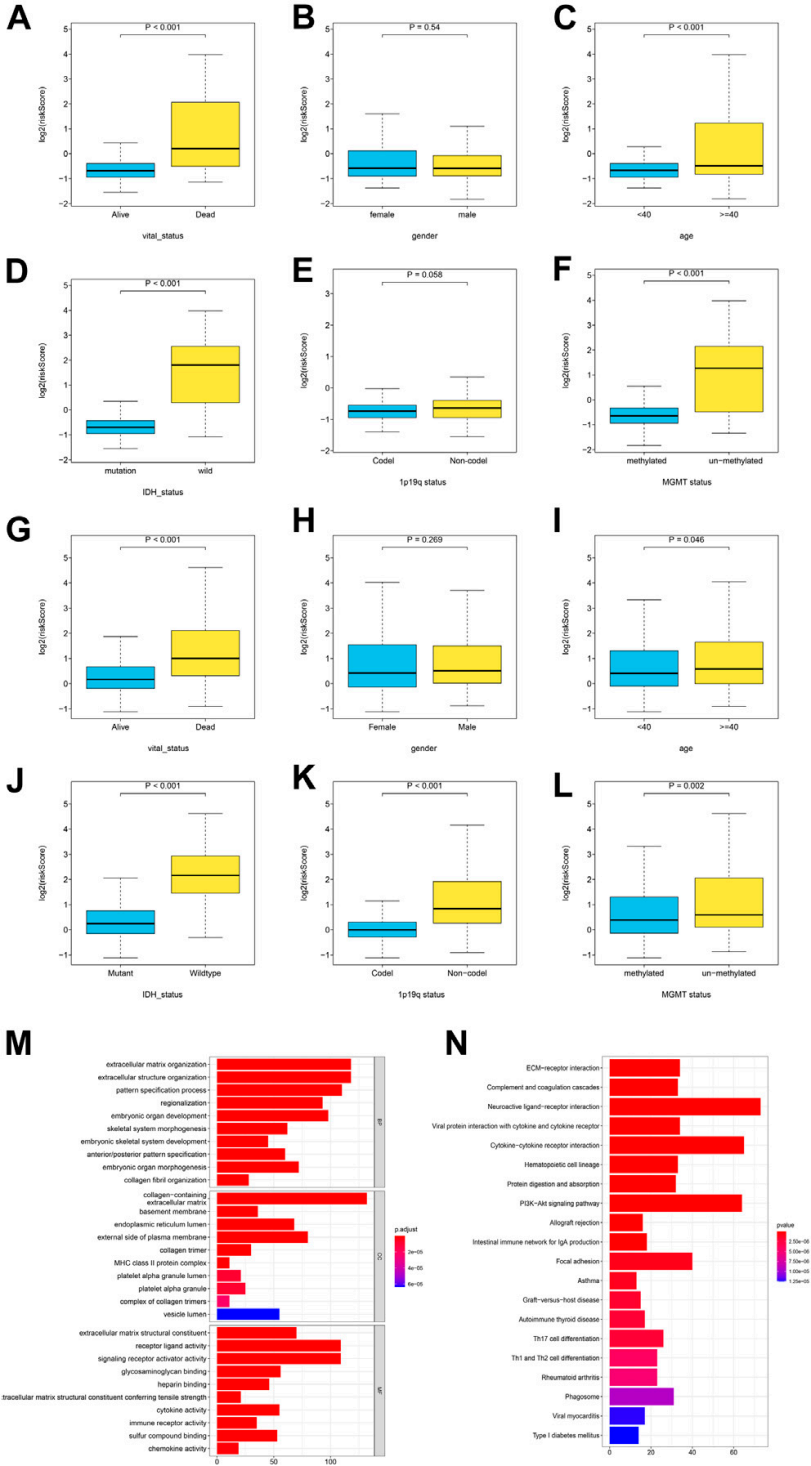
The correlation between the log2 (GRS score) and clinicopathological features and pathway enrichment analysis. **(A-F)** Distribution of GRS scores in different subgroups in TCGA cohort, and **(G-L)** CGGA cohort. **(M-N)** GO and KEGG enrichment analysis for differentially expressed genes between the high- and low- GRS score groups.

To identifying the relationship between the GRS score and the clinicopathological characteristics, we explored the corresponding clinical information of LGG cases in the TCGA training cohort and the CGGA testing cohort (The clinical information in the Rembrandt cohort was incomplete). The GRS score was associated significantly with age, survival status, and *IDH1* status. The results showed that the GRS score was high in “Dead (survival status)”, “> 40 (Age)”, *“IDH1* wild-type”, non-codel (1p19q status) unmethylated (MGMT status) group, the respective *p* values were below 0.05 (Figures 5A–F), the same trend was observed in the CGGA cohort (Figure 5G-L).

### Pathway Enrichment Analysis and Visualization of Differentially Expressed Gene (DEGs) Between the High and Low GRS Score Groups

To explore the association of gene expression with GRS score, gene expression in 501 patients with LGG in the training set was compared between the high and low GRS score groups, to identify differentially expressed genes (DEGs). We obtain 1827 differentially expressed genes (*p* < 0.05, |log_2_FC|>1), Gene ontology (GO), and Kyoto Encyclopedia of Genes and Genomes (KEGG) were performed on the DEGs. The GO analysis showed that a higher GRS score was associated with extracellular matrix organization and extracellular structure organization (Figure 5M). The KEGG analysis indicated that a higher GRS score was related to ECM−receptor interaction, Complement and coagulation cascades (Figure 5M). Taken together, these results suggested that the proteins encoded by the GRS genes have important functions in tumor microenvironment (TME) remodeling in patients with LGG.

### GRS Acts as an Indicator of Therapeutic Resistance and Potential Treatments for Patients With High GRS Score Patients

**FIGURE 6:**
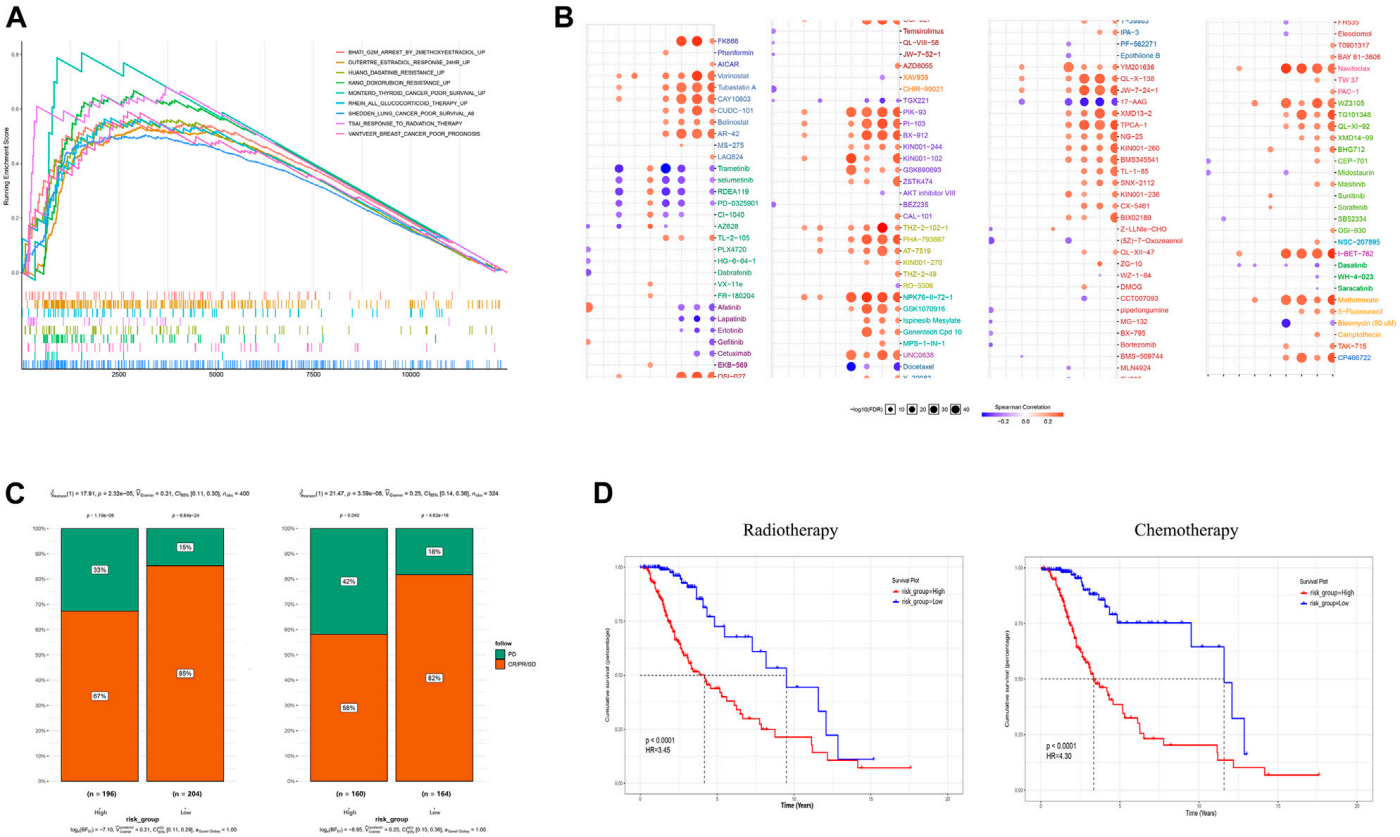
The gene signature is a good marker of resistance to various treatments. **(A)** A high GRS score correlated with therapeutic resistance. **(B)** A landscape graph illustrating the drug sensitivity (IC50) of members of the GRS signature in LGG cells. **(C)** The clinical benefit rate (CBR) was lower in the high GRS score groups both in follow-up treatment outcome terms and primary treatment outcome terms. **(D)** Patients with a higher GRS score had a worse OS among those that received chemotherapy and radiotherapy. CBR: CR + PR + SD (CR, complete remission; PR, partial remission; SD, stable disease; PD, progressive disease) IC50, half maximal inhibitory concentration.

Tumor glycolysis increases therapy resistance; therefore, we determined if the GRS could function as an indicator of therapeutic resistance (Hamilton et al., 2018). In addition to being associated with poor survival, a higher GRS score correlated significantly with resistance to various therapies, such as targeted therapy, radiation therapy, and chemotherapy, according to GSEA in the training cohort (Figure 6A). The online tool GSCALite was used to draw landscape plot (bubble heatmap), which demonstrated the relationships between gene signature members and drug responses (Figure 6B). Genes in the signature correlated significantly with the half-maximal inhibitory concentration (IC50) data in LGG cells. The genes *ABCC3*, *RHBDF1*, and *PTGFRN* conferred drug resistance, which were consistent with the results shown in Figure 3E. Next, clinical outcomes and treatment information from the training cohort were used to verify these predictions. Following surgery, the clinical benefit rate (CR, complete remission; PR, partial remission; SD, stable disease) was remarkably lower (*p* < 0.0001) in the higher GRS group, both in terms of follow-up treatment outcome and primary treatment outcome (Figure 6C). Furthermore, among patients who had been treated with adjuvant therapies (chemotherapy and radiotherapy), patients with a higher GRS score had worse (*p* < 0.0001) OS than those with a lower GRS score (Figure 6D).

### Integrating the Glycolysis Signature With Clinicopathological Features to Improve Risk Stratification and Survival Prediction

**FIGURE 7:**
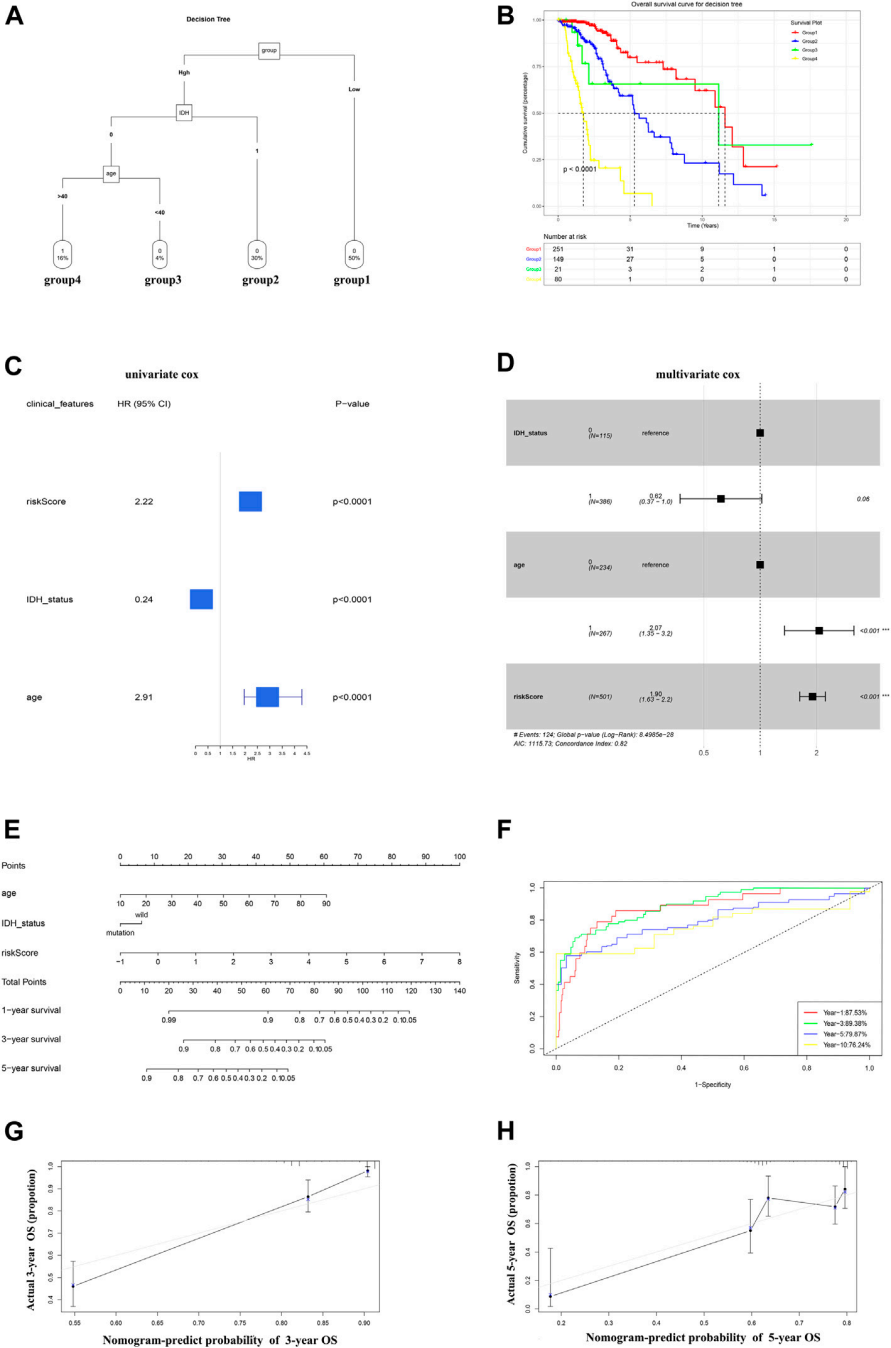
A nomogram and decision tree constructed on the basis of glycolysis and clinicopathological features in TCGA. **(A)** The decision tree that was established to increase the level of risk stratification. **(B)** Kaplan-Meier curves showing the OS of subgroups established using the decision tree. **(C-D)** Univariate cox (*p* < 0.05) and Multivariate Cox (*p* < 0.2) analysis of clinicopathological factors. **(E)** A nomogram was drawn to predict the survival rate at different time points for individual patients. **(F)** ROC curves at 1, 3, 5, and 10 years showing that the nomogram is a robust indicator for OS. **(G-H)** Three-year and 5-year calibration curves of the training cohort.

A decision tree was constructed to increase the stratification of risk using the three parameters available age (≥40 or <40), IDH status (wild and mutant), and the GRS (high and low), the results of which showed that all factors (nsplit = 3, xerror is minimum) remained in the decision tree and four different risk subgroups were identified (Figure 7A). The GRS score played an important role in the model. Kaplan–Meier curves showed that OS differed significantly among the four risk subgroups. Patients in the high GRS score, wild-type *IDH1* status, advanced age (age >40) subgroups had the highest risk (Figure 7B). The patients in the training cohort with age, sex, *IDH1* status, and GRS scores were used for further study. Univariate Cox regression analysis showed that age, *IDH1* status, and GRS score were associated significantly with the prognosis of LGG (Figure 7C, *p* < 0.05). Multivariate Cox regression analysis identified GRS as an independent prognostic factor combined with other clinicopathological factors (Figure 7D, *p* < 0.2). These three factors (The GRS score was scaled) were constructed into a nomogram to quantify the risk assessment and survival probability for individual patients with LGG (Figure 7E). The forecast curve of the calibration analysis (black line) of the nomogram for 3-years and 5-years survival probability closely resembled that of the ideal performance (the grey line in Figures 7G,H), indicating that the nomogram was highly accurate. Finally, the nomogram’s reliability was evaluated using time-dependent ROC curves (Figure 7F). The AUC values were 0.875, 0.893, 0.799, and 0.762 for 1-, 3-, 5-, and 10-years survival, respectively, indicating a good potential in clinical practice for monitoring survival. We conduct same analysis in the CGGA cohort, the result was exhibited in Supplementary Figure S3, which also suggest high reliability of our model.

## Discussion

Compared with normal cells, tumor cells have many unique hallmarks, which are of great significance in the precise treatment of tumors. Metabolic change is one of the most intriguing areas of tumor research (Pavlova and Thompson, 2016). To meet their high metabolism, tumor cells must struggle to get enough nutrients (Ou and Lv, 2020). More than 90 years ago, Warburg et al. found that tumor cells maintain active glycolysis levels even in the absence of oxygen. This landmark discovery is known as the “Warburg Effect” (Liberti and Locasale, 2016). Since then, the study of glucose metabolism in tumors has been in full swing. Positron emission tomography (PET) imaging based on cancer glucose metabolism has been widely used in clinical and plays an important role in the diagnosis and monitoring of tumors (Walker et al., 2020). Therapies targeting glucose metabolism, such as glucose transporter (GLUT) inhibitors, also hold great promise in the treatment of tumors (Reckzeh and Waldmann, 2020). The role of glucose metabolism in cancer is becoming increasingly attractive.

As research progressed, the role of glucose metabolism in tumors was found to be related to more than just energy supply. Some intermediates of glucose metabolism, such as lactic acid, may be involved in improving the tumor microenvironment and mediating immune reprogramming (Vaupel et al., 2019). Clinicians can also be able to choose and tailor treatments based on well-established disease models. Zhang et al. constructed immune infiltrating cells-derived risk signature, which can well describe immune characteristics and predict prognosis in glioma patients (Zhang et al., 2021). Therefore, the current research on glucose metabolism is far from enough. In addition, several clinical studies have found that high glucose metabolism is associated with poor prognosis in some tumors. Zhang et al. constructed a prognostic signature of glycolysis-related genes in lung adenocarcinoma (Zhang et al., 2019), in which patients in the high-risk group had a worse prognosis. Yu et al. also constructed a prognostic model of glycolysis-related genes in gastric cancer and found that glycolysis was related to prognosis and immune infiltration in gastric cancer patients (Yu et al., 2020). However, for the low-grade glioma, a tumor with a very poor prognosis, no glycolysis-related prognostic model has been established. Therefore, it is necessary to evaluate the prognostic significance and therapeutic guidance value of glycolysis in low-grade glioma.

In this study, using the ssGSEA algorithm and univariate regression model, glycolysis was identified as the primary OS-related risk factor in patients with LGG. Combining algorithm WGCNA, univariate Cox regression and LASSO Cox regression model, we obtained robust prognostic candidates and constructed a glycolysis-related gene signature (GRS). Next, the gene signature’s prognostic value was confirmed by multiple analyses in two other cohorts. The clinical characteristics of patients with LGG were analyzed to determine the correlation between the GRS score and clinicopathological features, which showed that the GRS score was elevated significantly in patients with older age (>40), wild-type *IDH1* status, and those who had died. Thereafter, GO, KEGG enrichment analysis based on DEGs between the high and low GRS score groups were carried out to explore what aspects of the high GRS score affected the TME of patients with LGG. In addition, patients with LGG with a high GRS score experienced poorer survival compared with those in the low GRS score group in the adjuvant therapy groups, which might have been caused by therapeutic resistance induced by members of the signature, which suggested that the GRS could also be used as a reliable tool to predict therapeutic resistance in patients with LGG. Finally, a decision tree was constructed to improve the stratification of risk, which was integrated with the clinicopathological characteristics. In the decision tree, the GRS score functioned as the major decisive factor. Meanwhile, the GRS was confirmed as an independent prognostic factor after adjusting for other clinicopathological features using multivariate Cox regression analysis. These results indicated that the GRS is a reliable risk factor for OS in patients with LGG. To increase the predictive ability of OS for LGG in a quantitative manner, a prognostic nomogram containing scale-GRS scores and other clinical features was established to predict the probability of 1-, 3-, 5-years OS. Calibration analysis demonstrated the accurate predictive ability of the nomogram, which was in accord with actual survival. Furthermore, ROC analysis demonstrated that the nomogram model exhibited high accuracy to predict survival in the timeline of follow up.

Some candidates included in the signature, have been reported to be involved in the glucose metabolic process of low-grade glioma cancer. ARL3 cycling between an in active GDP-bound and an active GTP-bound form, is involved in energy metabolic process, which may be associated with glucose metabolic process (Zhou et al., 2006; Veltel et al., 2008). Rahman et al. demonstrated that IGFBP2 could induce the increase of glioma invasion and malignancy by activating PTEN and AKT pathways, which is enhanced by HIF-α associated with promoting glycolysis related activities (Rahman and Thomas, 2011; Das et al., 2013). Li et al. reported that the express of RHBDF1 inhibited the RACK1 induced HIF-α degradation in breast cancer, which aggravated the hypoxic environment of the tumor environment and promoted the activation of the glycolysis pathway (Zhou et al., 2014). Based on comprehensive bioinformatic analysis, this study further screened some potentials which have not been reported in glucose metabolic process, which may provide new insight for the further research in glucose metabolic process of glioma.

Meanwhile, glycolysis related signature shows high accuracy in predicting the survival rate in 3 cohorts in our study, furthermore the signature displays strong correlation with clinicopathological features. All the results indicate GRS can serve as a reliable indicator in clinical application. To test the correctness of the model and improve the prediction ability of the model, more independent cohorts should be involved in our study. Go and KEGG analysis reveal fact glycolysis is a tumor hallmark involving gene networks (all the members of GRS) rather than some individual “glycolysis genes”, which is involved in tumor microenvironment remodeling. It gives us new enlightenment for the treatment of glioma which should be considered as a whole from multiple targets. In addition, patients with high GRS score exhibited poorer survival treated with traditional adjuvant therapy methods, which implicated urgent need for new means for treatment. we exhibited the drug sensitivity (IC50) of members of the GRS signature in LGG cells, which is need further biological experiments to validate the effectiveness *in vitro* and vivo.

At last, this study constructed a robust predictive model which can increase the abilities of risk stratification and survival prediction. However, the clinical information of cases in incorporated datasets were inconsistent. We established a predictive model based on the information available in TCGA training set, not including some important clinical features which can further improve the predictive ability of the model, such as p/19q status, WHO grade. In addition, the further verifications are needed by biological experiments and clinical studies.

## Conclusion

The present study has the advantages of incorporating different cohorts from the TCGA and CGGA databases to construct a robust glycolysis signature that can predict patient survival and therapeutic resistance with high accuracy in patients with LGG.

Combining the GRS signature with clinicopathological characteristics allowed us to construct a decision tree and a nomogram, which increased the abilities of risk stratification and survival prediction. The GRS might function as a reliable clinical prediction tool and might aid the future development of therapeutic targets.

## Data Availability

Publicly available datasets were analyzed in this study. This data can be found here: This study used online resources, which are available from the TCGA database at https://portal.gdc.cancer.gov/ and the CGGA database at http://www.cgga.org.cn/.
