# Supplementary figures and images for: Construction and Verification of a Glycolysis-Associated Gene Signature for the Prediction of Overall Survival in Low Grade Glioma
Source: Front Genet. 2022 Mar 23;13:843711. doi: 10.3389/fgene.2022.843711 (PMC8983898; doi:10.3389/fgene.2022.843711)

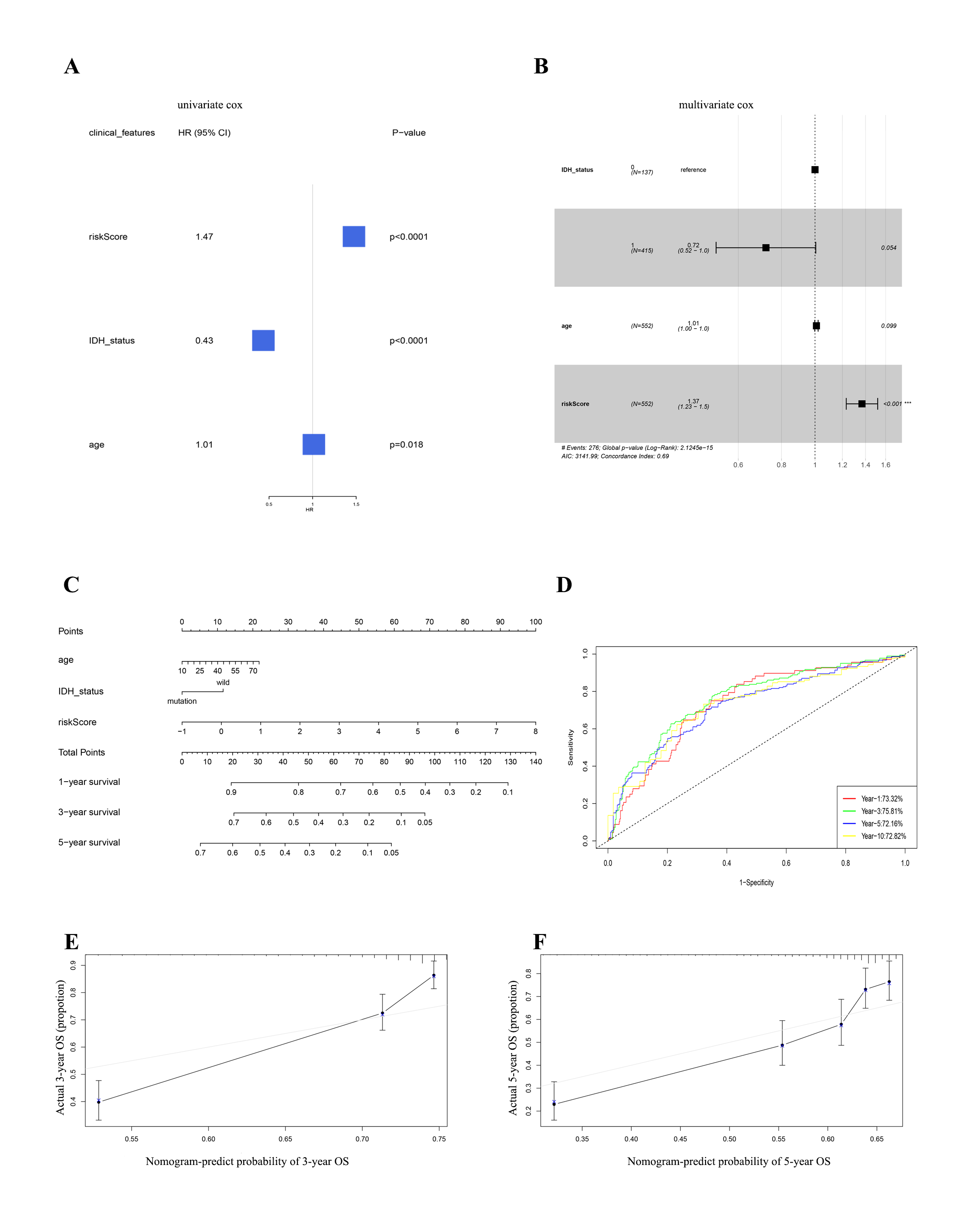

Supplement: Supplementary file 2 [file Image3.TIF]

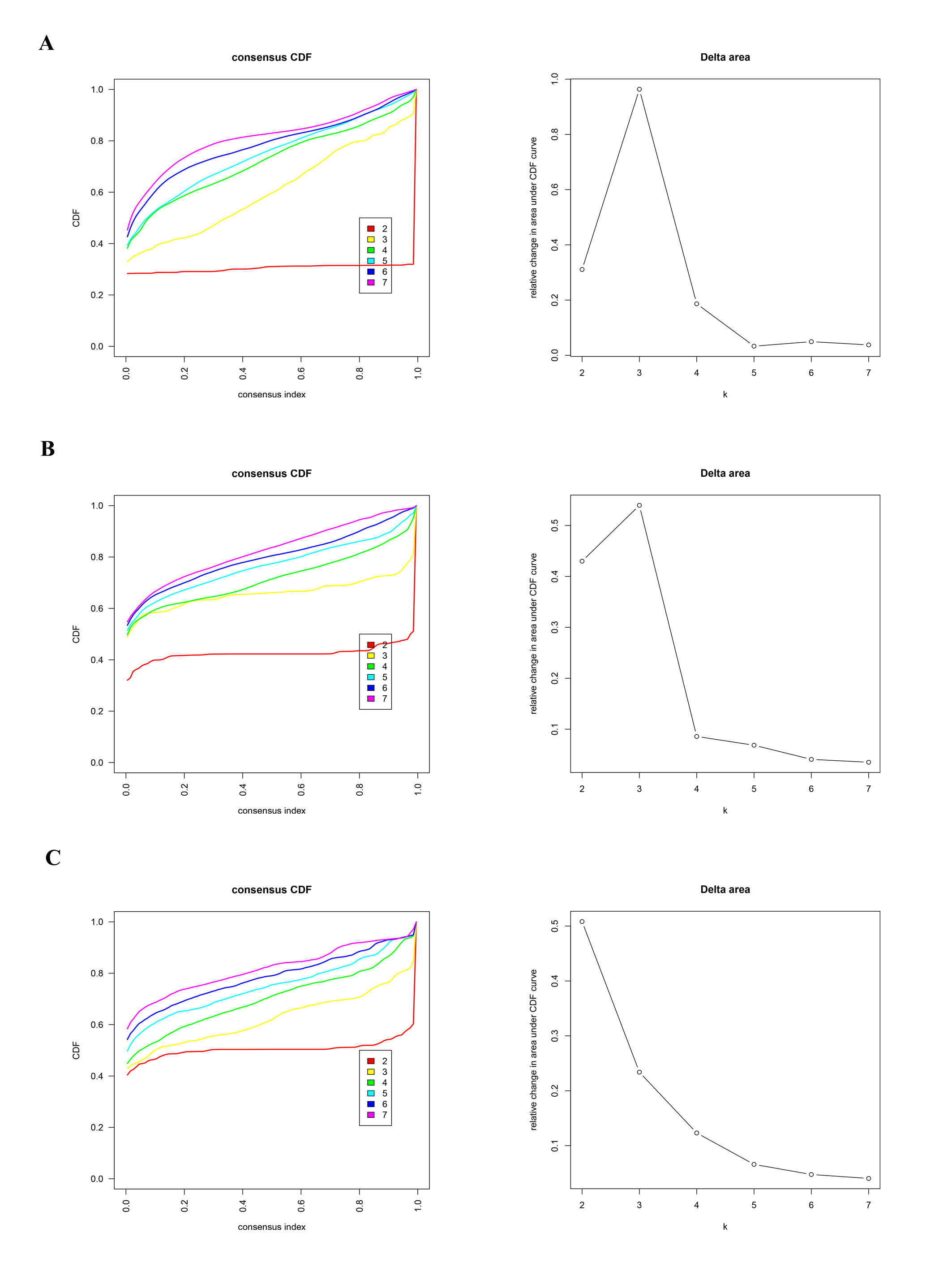

Supplement: Supplementary file 3 [file Image2.TIF]

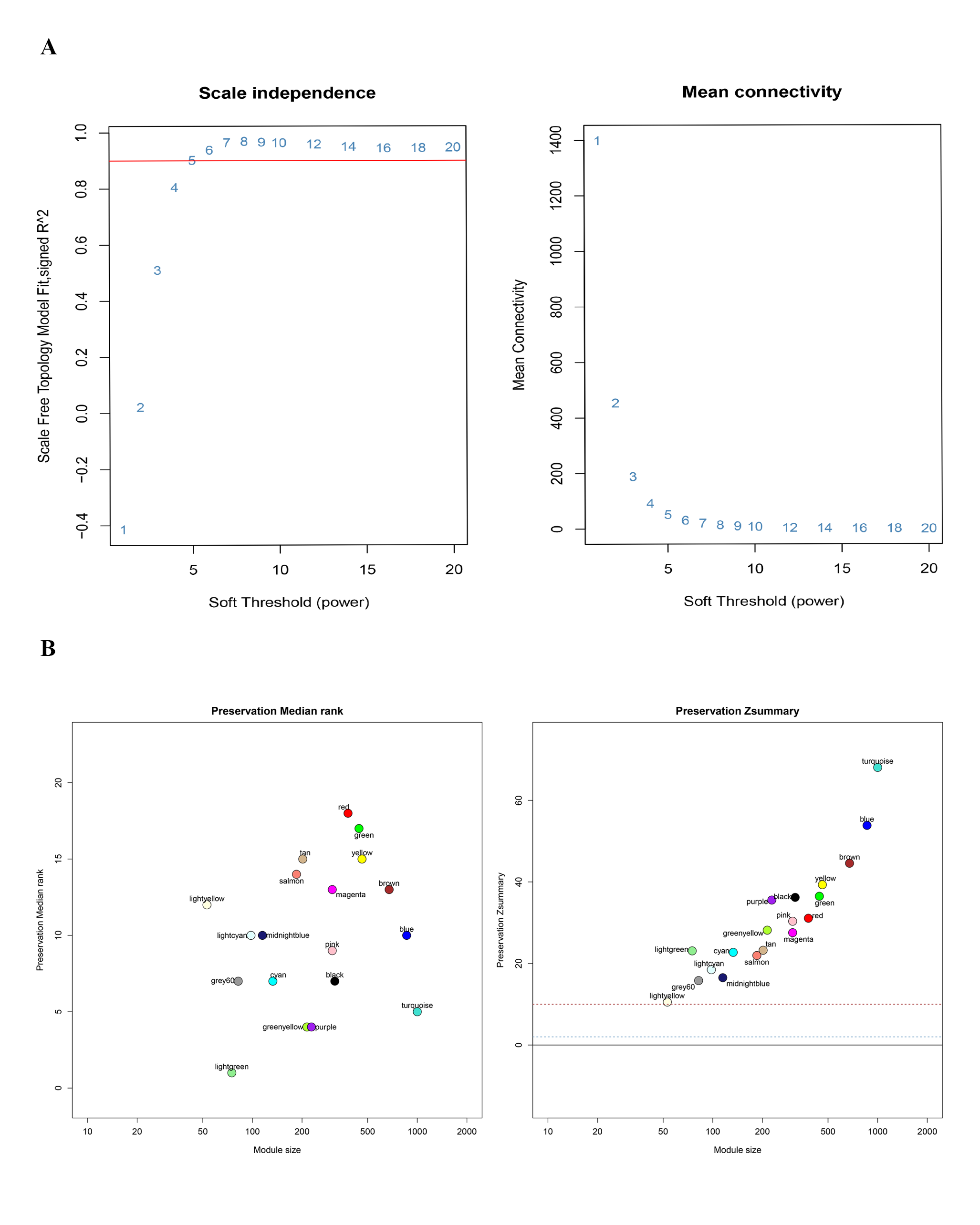

Supplement: Supplementary file 4 [file Image1.TIF]
